# Balloon-assisted coil embolization (BACE) of a wide-necked aneurysm of the inferior pancreaticoduodenal artery

**DOI:** 10.1186/s42155-020-00155-w

**Published:** 2020-09-05

**Authors:** Francesco Modestino, Alberta Cappelli, Cristina Mosconi, Giuliano Peta, Antonio Bruno, Giulio Vara, Caterina De Benedictis, Rita Golfieri

**Affiliations:** Department of Diagnostic Medicine and Prevention, Radiology Unit, S. Orsola-Malpighi Hospital, University of Bologna, Bologna, Italy

**Keywords:** Visceral aneurysm, Coils embolization, Inferior pancreaticoduodenal artery, Celiac trunk occlusion

## Abstract

**Background:**

Aneurysms of the pancreaticoduodenal arcades are an uncommon pathology, with a prevalence of 2%, and could be congenital or acquired. Treatment of visceral aneurysms is therefore generally recommended when the aneurysmal sac equals or exceeds 2 cm. Wide-necked (> 4 mm) and main artery branch aneurysms represent a challenge for conventional endovascular coil embolization due to the risk of coil migration.

**Main body:**

This case describes the technical feasibility of balloon-assisted coil embolization (BACE) in the treatment a wide neck aneurysm of inferior pancreatic duodenal artery due to celiac axis occlusion.

**Short conclusion:**

In case of celiac trunk occlusion, BACE is a safe procedure associated with optimal technical success rates, in order to treat the aneurysms and to preserve splanchnic vascularization.

## Introduction

Aneurysms of the pancreaticoduodenal arcades were described as early as late eighteenth century; true aneurysms of the pancreaticoduodenal arcades are rare and make up only 2% of all splanchnic aneurysms (Kalva et al. 2007). These may be congenital due to compression by the median arcuate ligament of celiac trunk or acquired secondary to fibromuscular dysplasia or atherosclerosis (Kalva et al. 2007).

The risk of rupture is low but increases significantly with enlarging aneurysm size, carrying a mortality rate of up to 80% (Kok et al. 2016). Treatment of visceral aneurysms is therefore generally recommended when the aneurysmal sac equals or exceeds 2 cm (Ibrahim et al. 2018). Despite that, the risk of rupture of pancreaticoduodenal arcade aneurysms is independent of the aneurysmal diameter (Nishiyama et al. 2013). Wide-necked (> 4 mm) and main artery branch aneurysms represent a challenge for conventional endovascular coil embolization due to the risk of coil migration (Ibrahim et al. 2018).

In the past, surgery was the only treatment for pancreaticoduodenal artery aneurysm. Recently, the development of interventional radiology has made possible to perform transcatheter arterial embolization of visceral aneurysms safely and effectively.

We describe a case of a wide neck of inferior pancreatic duodenal artery (iPDA) aneurysm associated with a celiac trunk occlusion treated with balloon-assisted coil embolization (BACE) in order to treat the aneurysms and to preserve retrograde celiac trunk vascularization through the pancreatic-duodenal arcade.

## Case report

**Fig. 1:**
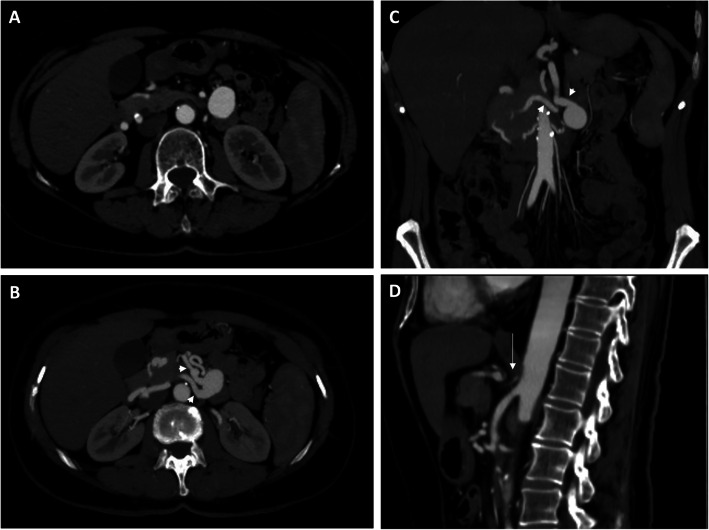
CT angiogram, performed in a 58 years-old woman with a history of abdominal pain and an occasional feedback of an aneurysm of the inferior pancreatic duodenal artery. The aneurysmal sac measured 2,6 × 2,1 cm in maximum diameter (**a**) (arrowheads). Maximum intensity projected reconstruction (**b**, **c**) better depicted the aneurysm morphology with relatively wide neck and dilated inferior pancreatic duodenal artery (arrowheads); the sagittal reconstruction showed celiac trunk occlusion (**d**, white arrow)

A 58 years-old woman with a history of recurrent abdominal pain, especially post prandial, and occasional episodes of diarrhoea, underwent a magnetic resonance enterography (MRE) that demonstrated a saccular vascularized formation near the aorta. Therefore, the patient underwent Computed Tomography (CT) angiogram that showed an aneurysm of the iPDA measuring 2,6 cm × 2,1 cm in maximum diameter, with a relatively wide aneurysm neck of 12 mm; maximum intensity projected reconstruction of the CT images revealed celiac trunk occlusion with dilated iPDA (Fig. 1).

In view of the aneurysm size, a decision was made in consensus with the patient for endovascular treatment following discussion at a multidisciplinary meeting.

Under local anaesthesia, a bilateral common femoral artery access was granted under ultrasound guidance. On the right a 7F guiding sheath (Mach1 Boston Scientific, Cork, Ireland) was advanced into the abdominal aorta while on the left side a 5F sheath (St. Jude Medical™USA) was positioned. We choose two groin punctures to have greater control of the devices and grant further access in case of complications.

**Fig. 2:**
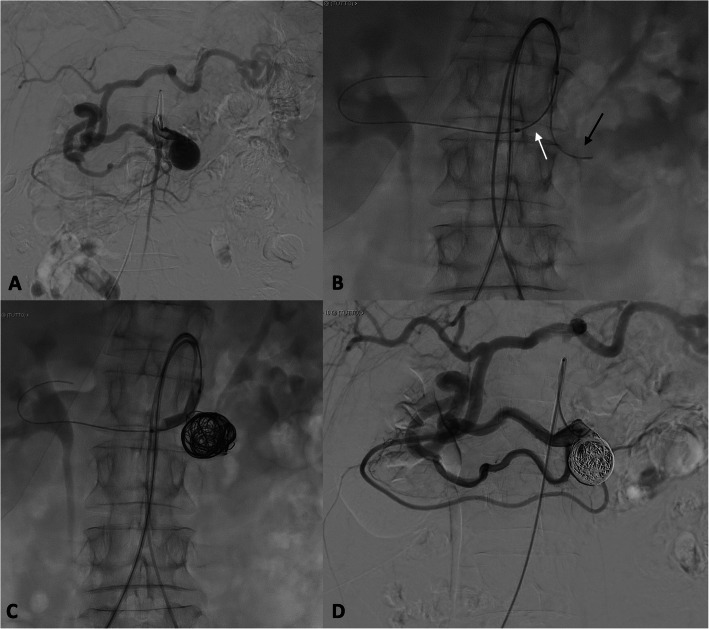
preliminary angiogram confirmed the aneurysm and dilated inferior pancreatic duodenal artery with evidence of revascularization through this branch of the celiac trunk (**a**). A balloon was positioned across aneurysm neck and the aneurysmal sac was catheterized with a microcatheter (**b**, black arrow). Then balloon was carefully inflated, and detachable non-fibered coils were delivered into the aneurysmal sac (**c**). The final diagnostic angiography showed completely exclusion of the sac from blood filling with preserved flow through PDA to the celiac axis

We tried unsuccessfully to catheterize the celiac trunk occlusion to treat the aneurysm. Then we decided to treat the aneurysmal sac through superior mesenteric artery (SMA). This latter was catheterized with 5F Cobra 2 catheter (Terumo, Tokyo, Japan) and a 0,0035″ angled guidewire (Terumo, Tokyo, Japan); subsequent DSA (digital subtraction angiography) obtained from the origin of SMA angiogram confirmed the saccular aneurysm, dilated iPDA with evidence of revascularization through this branch of the celiac trunk (Fig. 2a).

In order to avoid iPDA embolization and preserve celiac branches, a 6 × 40 mm balloon (Mustang™, Boston Scientific, Cork, Ireland), sized on the basis of CT images, was advanced through the right access and positioned across aneurysm neck; then across the left side access, we catheterized the aneurysmal sac with a microcatheter (2.7 F tip Progreat®; Terumo, Tokyo, Japan) (Fig. 2b). Following administration of 3000 units of heparin, the balloon was carefully inflated to low pressure under fluoroscopic control; after balloon inflation, 5 detachable non-fibered coils, 20 mm × 50 cm, (Concerto™ Detachable Coil System, Medtronic) were delivered to pack the aneurysmal sac until its complete filling (Fig. 2c).

Then, balloon was deflated and a diagnostic angiography was performed that showed completely exclusion of the sac from blood filling with preserved flow through PDA to the celiac axis (Fig. 2d).

Haemostasis was obtained with closure device 8F (AngioSeal® Vip Vascular Vip closure devise) on the right and manual compression on the left side.

There were no immediate peri-procedural complications and patient was discharged after 24H.

**Fig. 3:**
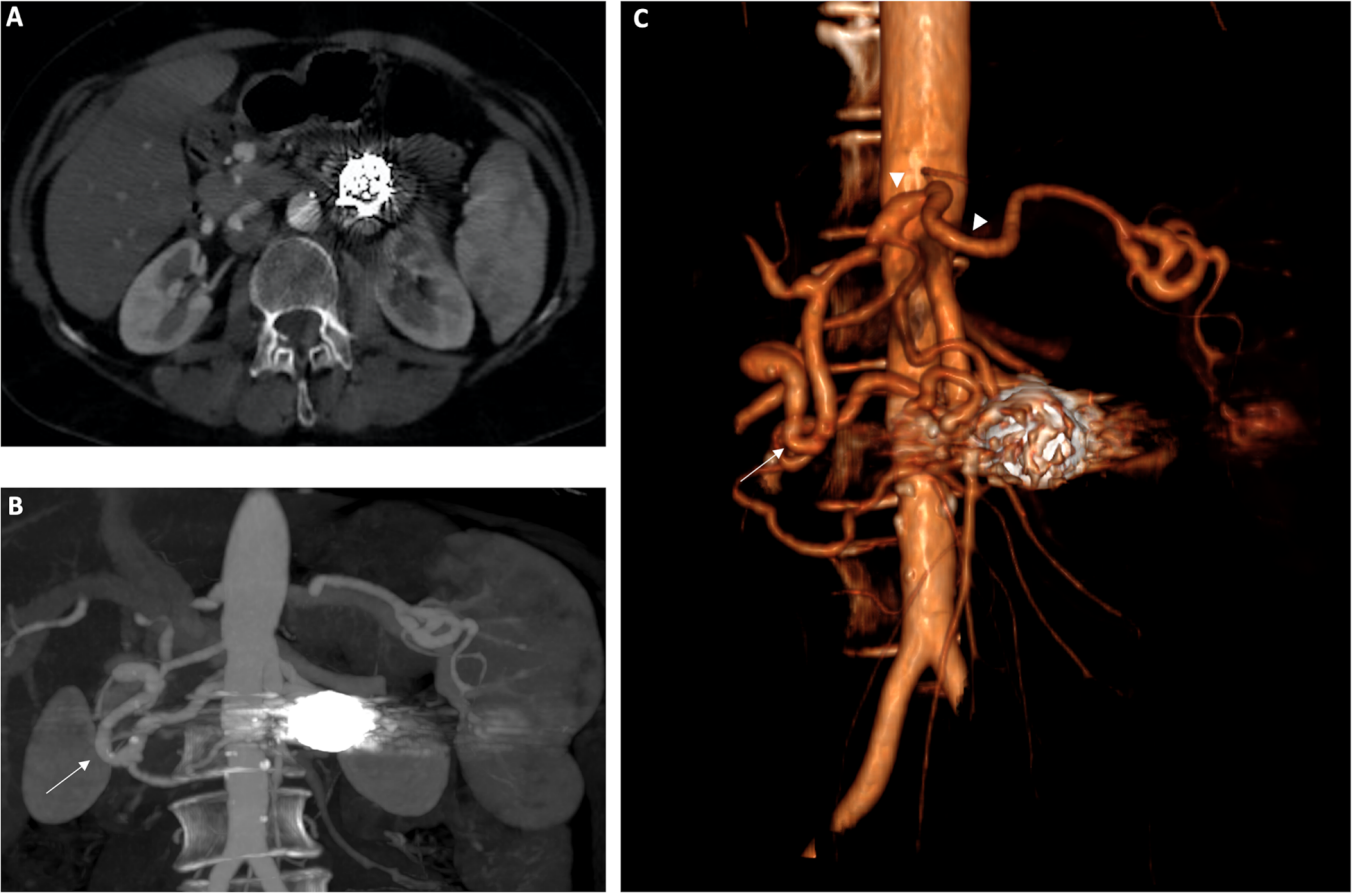
CT at 12 months showed complete exclusion of the aneurysmal sac from blood (**a**) flow with patency of inferior pancreatic duodenal artery and preserved vascularization of celiac branches (**b**, white arrow)

The patient came back, as a standard of our Institute, for a follow-up abdominal CT at 3 and 12 months later to monitor the onset of complications, the maintenance of visceral flow and the persistence of our success. Both CT images confirmed complete exclusion of the aneurysmal sac from blood flow with patency of iPDA and preserved vascularization of celiac branches (Fig. 3).

## Discussion

True aneurysms of the pancreaticoduodenal arcades are seen in the setting of celiac occlusion with hepatic perfusion from the pancreaticoduodenal arcades/gastroduodenal artery. The pathophysiology of these aneurysms is poorly explained, but one hypothesis could be that increased flow through the small, fragile pancreaticoduodenal arteries in the presence of celiac axis stenosis/occlusion may be responsible of the formation of the aneurysms. The vessels enlarge to accommodate the increased flow. In a few cases, the persistent increased flow and high intraarterial pressure lead to weakening of the vessel wall and the formation of a true aneurysm (Kobayashi et al. 2004; Kallamadi et al. 2009).

Pancreaticoduodenal artery aneurysm ruptures can be a life-threatening emergency. Before 1980, surgery was the only treatment for pancreaticoduodenal artery aneurysm. More recently the interventional radiology techniques allowed a safe and effective endovascular approach to treat visceral aneurysms. Coll et al. (Coll et al. 1998) reported that, since 1980, the mortality rate associated with surgery has been 19%, whereas that associated with transcatheter arterial embolization has been 0% (Flood and Nicholson 2013). Subsequently, some researchers have reported that transcatheter arterial embolization is effective in the treatment of visceral aneurysms, showing few complications and low recurrence rate (Ibrahim et al. 2018).

The embolization of wide-necked aneurysm has been described using flow diversion techniques, covered stents, neurovascular devices, involved vessel embolization and BACE (Ibrahim et al. 2018; Venturini et al. 2017). We selected BACE as the preferred technique in this case both to immediately occlude the aneurysm and to preserve the retrograde celiac trunk vascularization.

Some authors reported that, in cases of iPDA aneurysm with celiac stenosis, it may be better to simultaneously treat both conditions with endovascular treatment, if possible (Venturini et al. 2017). In the case of celiac axis stenosis or occlusion in which pancreaticoduodenal artery aneurysms cannot be selectively embolized, some authors reported that transcatheter arterial embolization without bypass may lead to recurrence of pancreaticoduodenal artery aneurysm or ischemic injury as a result of the absence of major collateral vessels (Murata et al. 2006). However this point still appears controversial; in our case we decided not to treat the obstruction and 12 months later, there was no presence of new aneurysmal features.

However, BACE presents several limitations, such as increased operative complexity owing to greater number of guidewires and microcatheters required intra-procedurally and vessel dissection or rupture secondary to inflation of the balloon microcatheter near the aneurysm neck. Therefore, it is recommended to perform this approach in centre with a large radiologic interventional experience.

## Conclusions

In conclusion, BACE is a safe endovascular treatment of aneurysms of the pancreatic duodenal arteries associated with an optimal technical success rates, visceral flow preservation and a low rate of major complications.

## Data Availability

Data availability statement is not applicable.

## References

[CR1] Coll DP, Ierardi R, Kerstein MD, Yost S, Wilson A, Matsumoto T (1998). Aneurysms of the pancreaticoduodenal arteries: a change in management. Ann Vasc Surg.

[CR2] Flood K, Nicholson AA (2013). Inferior pancreaticoduodenal artery aneurysms associated with occlusive lesions of the celiac axis: diagnosis, treatment options, outcomes, and review of the literature. Cardiovasc Intervent Radiol.

[CR3] Ibrahim F, Dunn J, Rundback J, Pellerito J, Galmer A (2018). Visceral artery aneurysms: diagnosis, surveillance, and treatment. Curr Treat Options Cardiovasc Med.

[CR4] Kallamadi R, Demoya MA, Kalva SP (2009). Inferior pancreaticoduodenal artery aneurysms in association with celiac stenosis/occlusion. Semin Intervent Radiol.

[CR5] Kalva SP, Athanasoulis CA, Greenfield AJ (2007). Inferior pancreaticoduodenal artery aneurysms in association with celiac axis stenosis or occlusion. Eur J Vasc Endovasc Surg.

[CR6] Kobayashi T, Uenoyama S, Isogai S (2004). Successful transcatheter arterial embolization of an inferior pancreaticoduodenal artery aneurysm associated with celiac axis stenosis. J Gastroenterol Hepatol.

[CR7] Kok HK, Asadi H, Sheehan M, Given MF, Lee MJ (2016). Systematic review and single-center experience for endovascular Management of Visceral and Renal Artery Aneurysms. J Vasc Interv Radiol.

[CR8] Murata S, Tajima H, Fukunaga T (2006). Management of pancreaticoduodenal artery aneurysms: results of superselective transcatheter embolization. AJR Am J Roentgenol.

[CR9] Nishiyama A, Hoshina K, Hosaka A, Okamoto H, Shigematsu K, Miyata T (2013). Treatment strategies for a pancreaticoduodenal artery aneurysm with or without a celiac trunk occlusive lesion Ayako Nishiyama. Ann Vasc Dis.

[CR10] Venturini M, Marra P, Colombo M (2017). Endovascular treatment of visceral artery aneurysm and pseudoaneurysm in 100 patients: covered stenting vs transcatheter embolization. J Endovasc Ther.

